# Secure Signal Encryption in IoT and 5G/6G Networks via Bio-Inspired Optimization of Sprott Chaotic Oscillator Synchronization

**DOI:** 10.3390/e28010030

**Published:** 2025-12-26

**Authors:** Fouzia Maamri, Hanane Djellab, Sofiane Bououden, Farouk Boumehrez, Abdelhakim Sahour, Mohamad A. Alawad, Ilyes Boulkaibet, Yazeed Alkhrijah

**Affiliations:** 1 Department of Electrical Engineering, Faculty of Sciences and Technologies, Abbes Laghrour University, Khenchela 40000, Algeriaboumehrez.farouk@univ-khenchela.dz (F.B.);; 2 Laboratory of Systems and Applications of Information and Communication Technologies (SATIT), Faculty of Sciences and Technologies, Abbes Laghrour University, Khenchela 40000, Algeria; 3 Department of Electrical Engineering, Faculty of Sciences and Technologies, Echahid Cheikh Larbi Tebessi University, Tebessa 12000, Algeria; 4 Laboratory of Signals and Smart Systems (L3S), Faculty of Sciences and Technologies, Echahid Cheikh Larbi Tebessi University, Tebessa 12000, Algeria; 5 Laboratory of Telecommunications (LT), Telecommunications Institute, 8 May 1945 University, Guelma 24000, Algeria; 6 Department of Electrical Engineering, College of Engineering, Imam Mohammad Ibn Saud Islamic University, Riyadh 11432, Saudi Arabia; 7 College of Engineering and Technology, American University of the Middle East, Egaila 54200, Kuwait

**Keywords:** IoT, 5G/6G networks, chaos-based encryption, Sprott chaotic oscillator, synchronization, Pachycondyla Apicalis Algorithm (API), Penguin Search Optimization Algorithm (PeSOA)

## Abstract

The rapid growth of Internet of Things (IoT) devices and the emergence of 5G/6G networks have created major challenges in secure and reliable data transmission. Traditional cryptographic algorithms, while robust, often suffer from high computational complexity and latency, making them less suitable for large-scale, real-time applications. This paper proposes a chaos-based encryption framework that uses the Sprott chaotic oscillator to generate secure and unpredictable signals for encryption. To achieve accurate synchronization between the transmitter and the receiver, two bio-inspired metaheuristic algorithms—the Pachycondyla Apicalis Algorithm (API) and the Penguin Search Optimization Algorithm (PeSOA)—are employed to identify the optimal control parameters of the Sprott system. This optimization improves synchronization accuracy and reduces computational overhead. Simulation results show that PeSOA-based synchronization outperforms API in convergence speed and Root Mean Square Error (RMSE). The proposed framework provides robust, scalable, and low-latency encryption for IoT and 5G/6G networks, where massive connectivity and real-time data protection are essential.

## 1. Introduction

The rapid expansion of Internet of Things (IoT) deployments and the growing diversity of modern communication infrastructures have generated a strong demand for lightweight and reliable security mechanisms. Many IoT nodes operate under stringent energy and computational constraints, which limits their ability to implement conventional cryptographic algorithms efficiently. As a result, alternative approaches that offer security with reduced computational overhead have attracted significant research interest.

Chaos-based secure communication is one such direction. Traditional cryptographic algorithms, including the Advanced Encryption Standard (AES) [1], the Data Encryption Standard (DES) [2], and the International Data Encryption Algorithm (IDEA), provide well-established security guarantees. However, their implementation on low-power IoT platforms may incur non-negligible latency and resource usage, especially when the protected data streams exhibit high redundancy or strong temporal correlation. This limitation has encouraged the exploration of complementary techniques based on classical encryption [3,4,5], neural networks [6], information theory [7], evolutionary computing [8], artificial intelligence [9,10], and, in particular, chaos theory [11,12,13,14].

Since the pioneering work of Pecora and Carroll [15], chaotic synchronization has been widely investigated for secure communication [16]. With the availability of low-cost embedded platforms, recent studies have demonstrated real-time implementations of chaotic oscillators and adaptive synchronization controllers [17,18]. Among the various chaotic models, the Sprott oscillator stands out for its mathematical simplicity, low computational cost, and rich dynamical behavior [19], making it an attractive candidate for lightweight physical-layer security in IoT systems.

A central requirement in synchronization-based secure communication is achieving fast and accurate convergence between the master and slave oscillators. Many existing synchronization schemes rely on adaptive or multi-loop controllers, which may increase implementation complexity and reduce practical feasibility in constrained IoT environments [20]. To address these challenges, this work introduces a synchronization-based chaotic communication framework using the Sprott oscillator, in which the intrinsic parameters and coupling gains are jointly optimized using two bio-inspired metaheuristic algorithms: the Pachycondyla Apicalis Algorithm (API) [21] and the Penguin Search Optimization Algorithm (PeSOA) [22]. These algorithms efficiently explore the parameter space and yield improved synchronization accuracy and faster convergence, even in the presence of noise and parameter uncertainty.

The main contributions of this work can be summarized as follows:

- A lightweight chaos-based secure communication framework tailored to the constraints of low-power IoT systems, relying on continuous-time synchronization of the Sprott oscillator.
- A unified joint-optimization strategy for estimating the intrinsic chaotic parameters (a,b,c) and the coupling gains $\left(k_{1},k_{2},k_{3}\right)$ using the API and PeSOA metaheuristic algorithms.
- Demonstration that the PeSOA-based optimization achieves faster convergence and lower synchronization error compared to API.
- Robustness evaluation under noisy channels and perturbed initial states, showing stable synchronization and reliable data recovery.
- A positioning of the proposed framework with respect to recent developments in chaos-based secure communication, highlighting its low complexity and practical applicability in resource-constrained environments.

The remainder of this paper is organized as follows. Section 2 reviews related work on chaotic systems, secure communication, and bio-inspired optimization. Section 3 describes the k-Sprott chaotic oscillator. Section 4 presents the synchronization and masking mechanism. Section 5 details the API and PeSOA optimization algorithms. Section 6 reports simulation results and comparative analyses. Finally, Section 7 concludes the paper and outlines directions for future research.

## 2. Related Work

Chaos theory has been widely investigated in secure communication due to its sensitivity to initial conditions, broadband spectral characteristics, and complex nonlinear dynamics. Early synchronization strategies, such as the adaptive scheme of Liao and Tsai [23], demonstrated that stable chaotic synchronization can effectively support secure communication channels. Hou et al. further examined the synchronization properties of Sprott systems and confirmed their suitability for chaotic masking in secure transmission scenarios [24].

Recent studies have increasingly incorporated intelligent and data-driven techniques to enhance synchronization performance. Zhang et al. proposed an RBFNN–PSO approach capable of synchronizing the Sprott B system under external noise, achieving robustness for image-encryption tasks [25]. Yau et al. used particle swarm optimization to perform chaotic synchronization in wireless environments, illustrating the feasibility of lightweight synchronization-based cryptosystems [26]. Liu and Zuo introduced a deep adaptive synchronization mechanism combining neural models with optimization strategies, enabling high-precision tracking of chaotic trajectories [27].

Bio-inspired and swarm intelligence techniques have also been explored in secure communication. Maamri et al. applied ant colony optimization for parameter estimation in Chua’s oscillator, demonstrating that metaheuristics can substantially improve synchronization accuracy [28]. Neurobiological principles have inspired additional synchronization controllers; for instance, Samimi et al. employed a Brain Emotional Learning (BEL) model to achieve chaos synchronization for secure data transmission [29].

Beyond single-loop strategies, multi-level and cooperative synchronization architectures have been proposed. Zourmba et al. introduced a multi-level synchronization scheme designed to enhance confidentiality in secure communication systems [30]. Dinu compared Lorenz and Rössler dynamics in cryptographic contexts, emphasizing the importance of selecting suitable chaotic attractors to ensure synchronization stability and security [31]. In a related direction, Özkurt applied interpretable AI techniques to analyze chaotic time-series in industrial robots, illustrating the potential of explainable models in complex dynamical environments [32].

Chaos-based communication has also been studied in cyber–physical systems. Chen et al. proposed a chaotic masking protocol that simultaneously provides secure communication and intrusion detection [33]. At the IoT networking level, several works have highlighted the challenges posed by constrained hardware platforms and emphasized the importance of lightweight security mechanisms. Alharby et al. investigated security–energy trade-offs in IoT nodes, underscoring the need for cryptographic schemes with reduced computational cost [34].

Despite these advances, prior work has not jointly optimized the intrinsic parameters of the Sprott oscillator together with its coupling gains using bio-inspired search strategies such as API and PeSOA. Moreover, previous studies have not examined optimization-driven synchronization within the specific constraints of IoT communication, where robustness and low computational complexity are often more critical than high throughput. The present study addresses these gaps by proposing a unified synchronization and optimization framework tailored to lightweight and resource-efficient secure communication.

## 3. K–Sprott Oscillator

Chaotic oscillators have attracted significant attention due to their rich nonlinear dynamics and their applicability in secure communications [35], system identification [36,37], control theory, and modern cryptographic frameworks [38]. Among the various chaotic systems proposed in the literature, the Sprott family of oscillators stands out for its structural simplicity, ease of hardware implementation, and ability to generate complex attractors even under minimal mathematical formulations [39,40].

J. C. Sprott introduced nineteen minimal chaotic flows, labeled alphabetically from A to S, each exhibiting distinct dynamical characteristics despite their compact structure [19]. In this work, we consider the K–Sprott oscillator, whose mathematical model is defined as follows:

(1) $$\begin{array}{cc} \dot{x} & =xy-az,\\ \dot{y} & =x-y,\\ \dot{z} & =bx+cz, \end{array}$$

where *a*, *b*, and *c* are real-valued system parameters that strongly influence the system dynamics.

**Figure 1:**
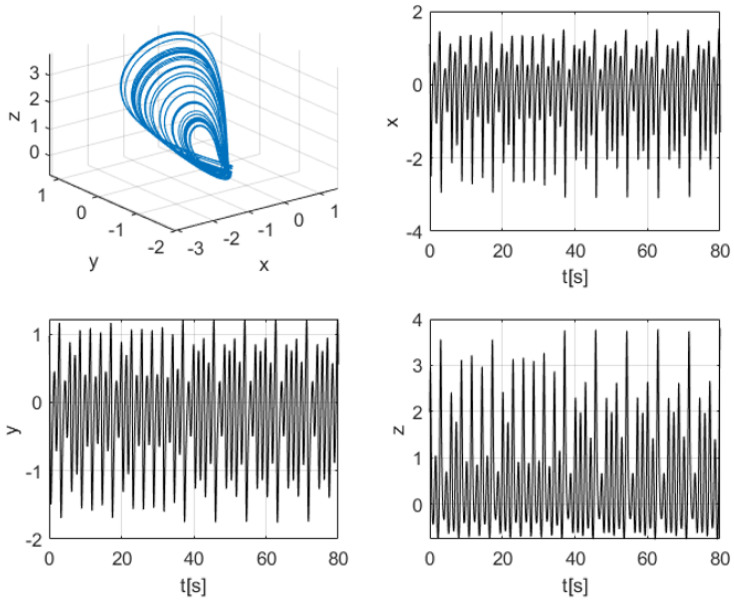
Chaotic behavior of the K–Sprott oscillator with a=1, b=1, and c=0.3. The blue curve represents the chaotic attractor, while the remaining panels show the time evolution of the state variables x(t), y(t), and z(t).

To illustrate the intrinsic dynamics of the K–Sprott system, Figure 1 presents the three-dimensional phase portrait and the corresponding time series of the state variables x(t), y(t), and z(t) for the representative parameter set a=1, b=1, and c=0.3. The resulting trajectories exhibit sensitive dependence on initial conditions and broadband oscillatory behavior, which are key properties that make Sprott systems suitable for secure communication and signal masking applications.

## 4. Encryption and Synchronization Process

In chaos-based secure communication, reliable transmission relies on establishing a stable functional relationship between two nonlinear dynamical systems. This mechanism, known as generalized synchronization, allows the receiver (slave) to reproduce the trajectory generated by the transmitter (master) [15]. Once synchronization is achieved, the masked information signal can be retrieved with high fidelity.

**Figure 2:**
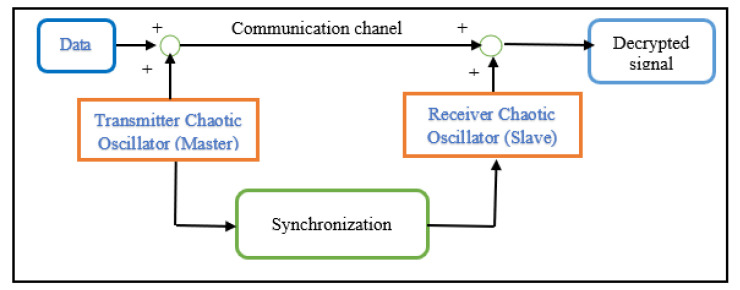
Chaotic masking scheme used for secure transmission and recovery.

Figure 2 illustrates the chaotic masking strategy considered in this work. The master Sprott oscillator generates a chaotic carrier, to which the information signal is added.

A slave oscillator with a similar structure attempts to track the master dynamics and recover the transmitted data after synchronization.

Synchronization is achieved using a diffusive full-state feedback controller:

(2) $$u\left(t\right)=K\left(x\left(t\right)-\hat{x}\left(t\right)\right),$$

where $K=[k_{1},\;k_{2},\;k_{3}]$. This control law injects the instantaneous state error into the slave dynamics and promotes convergence of the synchronization error when appropriate gains are selected.

We emphasize that the controller is not updated during the RK4 numerical integration. Instead, both the control gains $\left(k_{1},k_{2},k_{3}\right)$ and the intrinsic Sprott parameters (a,b,c) are jointly optimized at each iteration of the API or PeSOA metaheuristic algorithm. During each iteration, a candidate parameter vector is evaluated by integrating the master–slave Sprott systems with RK4 and computing the long-term synchronization error.

The master system is governed by the nonlinear dynamics

(3) $$\dot{x}\left(t\right)=f\left(x(t),\alpha \right),$$

where $x\left(t\right)={\left[x\left(t\right),\;y\left(t\right),\;z\left(t\right)\right]}^{T}$ is the state vector and α=(a,b,c) denotes the intrinsic parameters of the Sprott system.

The receiver (slave) system follows the same structure but includes an external control input:

(4) $$\dot{\hat{x}}\left(t\right)=f\left(\hat{x}\left(t\right),\alpha \right)+u\left(t\right).$$

To ensure synchronization between the two chaotic systems, a diffusive full-state feedback controller is applied:

(5) $$u\left(t\right)=K\left(x\left(t\right)-\hat{x}\left(t\right)\right),$$

where $K=[k_{1},\;k_{2},\;k_{3}]$ is the vector of control gains. Because the Sprott oscillator is third-order, the synchronization error involves three state components. The controller injects these errors into the slave dynamics and drives the error toward convergence when the gains are properly chosen.

The synchronization error is defined as

(6) $$e\left(t\right)=∥x\left(t\right)-\hat{x}\left(t\right)∥,$$

and its evolution is governed by

(7) $$\dot{e}\left(t\right)=f\left(x(t),\alpha \right)-f\left(\hat{x}\left(t\right),\alpha \right)-K\;e\left(t\right).$$

The fourth-order Runge–Kutta (RK4) scheme is used solely to integrate the master–slave Sprott system for any candidate parameter set. Parameter tuning is not performed through numerical integration but exclusively through two bio-inspired optimization algorithms, API and PeSOA. Both methods operate on a unified decision vector

$$\Theta =[a,\;b,\;c,\;k_{1},\;k_{2},\;k_{3}],$$

which gathers the intrinsic parameters and the diffusive control gains.

For each candidate Θ, the master and slave oscillators are simulated using RK4, and synchronization accuracy is evaluated through a long-term error-based cost function. The metaheuristics iteratively update Θ to minimize this cost, jointly estimating the intrinsic parameters and the coupling gains required to ensure complete synchronization and correct recovery of the masked signal.

**Figure 3:**
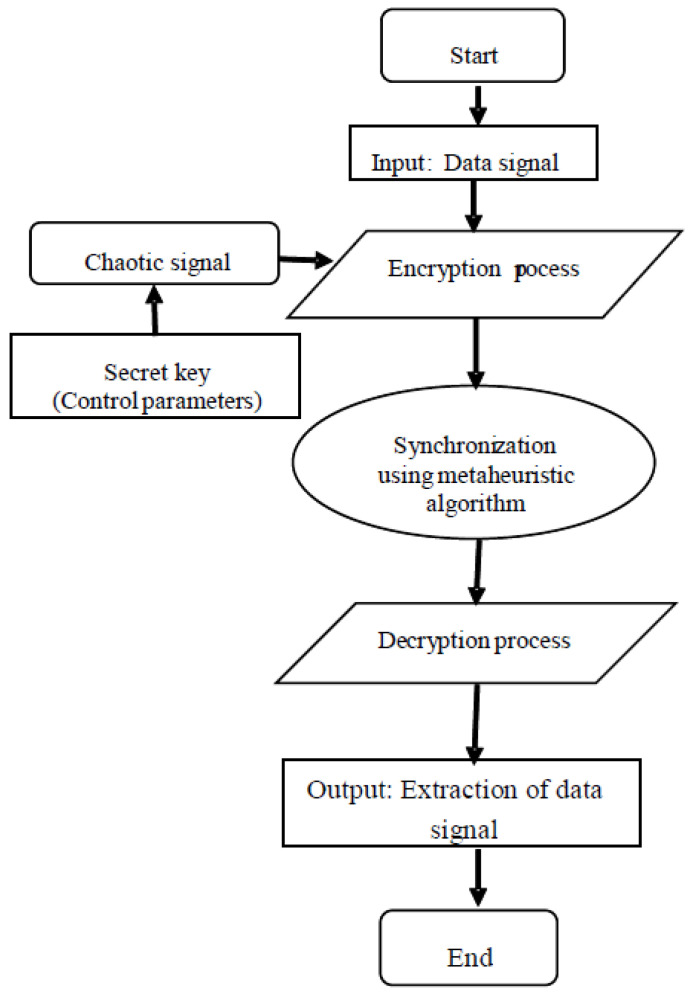
Flowchart of the proposed chaotic synchronization and secure communication mechanism.

Figure 3 summarizes the overall communication process, where the optimization stage precedes message reconstruction to ensure fast and reliable synchronization.

The synchronization performance is quantified using the cost function

(8) $$J=\frac{1}{N}\sum_{k=1}^{N}\sqrt{{\left(x\left(k\right)-\hat{x}\left(k\right)\right)}^{2}+{\left(y\left(k\right)-\hat{y}\left(k\right)\right)}^{2}+{\left(z\left(k\right)-\hat{z}\left(k\right)\right)}^{2}},$$

which provides a uniform measure of convergence across all state variables.

Table 1 summarizes the admissible parameter ranges of the Sprott system and the integration step used in the simulations. These ranges ensure chaotic behavior while enabling stable numerical integration.

## 5. Optimization-Based Synchronization and Stability Analysis

### 5.1. Pachycondyla Apicalis Algorithm (API)

**Figure 4:**
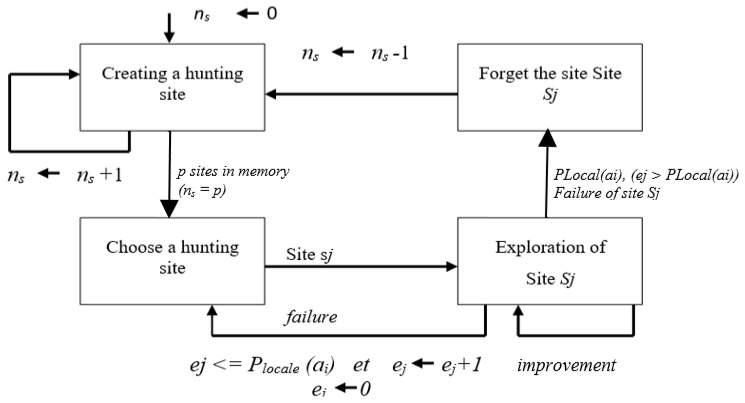
Exploration mechanism in the Pachycondyla Apicalis (API) algorithm.

The Pachycondyla Apicalis (API) algorithm is a bio-inspired optimization method derived from the foraging strategy of the ant species *Pachycondyla apicalis*. This algorithm was originally introduced by Monmarché, Venturini, and Slimane, who demonstrated that the ants’ hunting-site exploration and abandonment mechanism can be translated into an efficient search procedure [41]. In their model, each ant maintains a set of hunting sites around the nest and repeatedly explores, evaluates, and updates these sites according to their success. Figure 4 provides a simplified illustration of this exploration principle.

In the context of optimization, each ant represents a potential solution. During the search process, ants explore their neighborhood, retain promising hunting sites, and discard those that repeatedly fail. When all ants agree that the current nest position is no longer optimal, the nest is relocated to the best site found so far, enabling global exploration while preserving local improvement capacities.

The overall global foraging strategy is summarized in Algorithm 1, while the local exploration behavior of each ant is detailed in Algorithm 2.
**Algorithm 1** Global Foraging Strategy of the API Algorithm1:Initialize the nest position N←Orand2:T←0                            ▹ Iteration counter3:**while** stopping condition not met **do**4:      **for** each ant $a_{i}\in A$ **do**5:            Perform local exploration: API ← Foraging($a_{i}$)6:      **end for**7:      **if** nest relocation condition is satisfied **then**8:            $N\leftarrow S^{+}$                  ▹ Move the nest to the best site9:            Reset ant memories10:    **end if**11:    T←T+112:**end while**13:**return** best solution $S^{+}$ and its evaluation $f(S^{+})$

**Algorithm 2** Local Foraging Behavior of an Ant
1:**if** $nc(a_{i})<p$ **then**2:      Increment memory counter: $nc\left(a_{i}\right)\leftarrow nc\left(a_{i}\right)+1$3:      Create a new hunting site near the nest4:      $S_{nc}\left(a_{i}\right)\leftarrow \Theta_{expo}\left(N,A_{site}\right)$5:      $en(a_{i})\leftarrow 0$                      ▹ Failure counter6:
**end if**
7:Let $s_{j}$ be the last visited site8:**if** $e_{j}>0$ **then**9:      Randomly choose another site from memory10:
**end if**
11:Perform local exploration: $S_{u}\leftarrow \Theta_{local}\left(s_{j},A_{local}\right)$12:**if** $f\left(S_{u}\right)>f\left(s_{j}\right)$ **then**13:      Update site: $s_{j}\leftarrow S_{u}$14:      $e_{j}\leftarrow 0$15:
**else**
16:      Increment failure counter: $e_{j}\leftarrow e_{j}+1$17:      **if** $e_{j}>P_{local}$ **then**18:           Remove $s_{j}$ from memory19:           $nc\left(a_{i}\right)\leftarrow nc\left(a_{i}\right)-1$20:      **end if**21:
**end if**



### 5.2. Penguin Search Optimization Algorithm (PeSOA)

The Penguin Search Optimization Algorithm (PeSOA) is a bio-inspired metaheuristic that models the cooperative foraging behavior of penguin colonies. In nature, penguins dive in groups, adjust their trajectories according to previously successful dives, and communicate information about profitable feeding zones. PeSOA translates this collective strategy into an optimization process where each penguin represents a candidate solution exploring a specific region of the search space.The PeSOA algorithm imitates the foraging behavior of penguins [36,42].

At initialization, the population is divided into several subgroups, each assigned to a distinct exploration area. Within each subgroup, penguins update their positions using three main mechanisms: (i) local improvement based on their previous dive, (ii) movement toward the best solution found by the subgroup, and (iii) random perturbations to preserve exploration capability. An energy-like variable, referred to as the *oxygen reserve*, limits the number of consecutive dives and naturally balances exploration and exploitation. When a candidate solution improves its fitness, the penguin replenishes oxygen and continues exploring; otherwise, oxygen decreases until a new dive strategy is required. Throughout the search, the global best solution is continuously updated as penguins share information across groups. The procedure terminates when either the maximum number of iterations is reached or the convergence criterion is satisfied, as described in Algorithm 3.
**Algorithm 3** Penguin Foraging Strategy (PeSOA)1:Randomly initialize a population *P* divided into several groups2:Assign an oxygen reserve $O(p_{i})>0$ to each penguin $p_{i}$3:Set global best solution *G*4:**for** each $p_{i}\in P$ **with** $O(p_{i})>0$ **do**5:      $D_{\mathrm{new}}\leftarrow D_{\mathrm{last}}+\mathrm{rand}\left(\right)\cdot \left|X_{\mathrm{LocalBest}}-X_{\mathrm{LocalLast}}\right|$6:      Evaluate $f(D_{\mathrm{new}})$ and update $O\left(p_{i}\right)\leftarrow O\left(p_{i}\right)-1$7:      **if** $f\left(D_{\mathrm{new}}\right)>f\left(X_{\mathrm{LocalBest}}\right)$ **then**8:            $X_{\mathrm{LocalBest}}\leftarrow D_{\mathrm{new}}$9:      **else if** $f\left(X_{\mathrm{LocalBest}}\right)>f\left(G\right)$ **then**10:          $G\leftarrow X_{\mathrm{LocalBest}}$11:    **end if**12:**end for**13:**if** oxygen depleted or maximum iterations reached **then**14:      **Stop**15:**end if**

### 5.3. Lyapunov Stability Analysis

The reliability of the proposed synchronization-based secure communication scheme depends directly on the stability of the error dynamics between the master and slave K–Sprott oscillators. In the following, Lyapunov theory is employed in two complementary ways: (i) to verify that the master oscillator operates in a chaotic regime suitable for encryption, and (ii) to demonstrate the stability of the synchronization process required for successful decryption.

**Figure 5:**
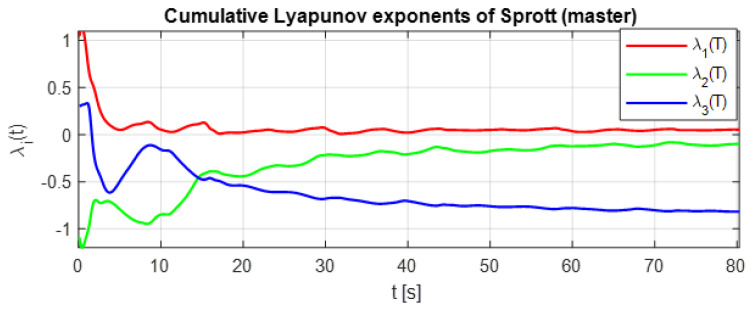
Lyapunov exponents of the K–Sprott system for the optimized parameters.

Figure 5 presents the cumulative Lyapunov exponents of the master K–Sprott attractor over an 80 s simulation interval. The spectrum exhibits one strictly positive exponent, one approximately zero exponent, and one negative exponent. This (+,0,−) configuration is the classical hallmark of deterministic chaos, confirming that the K–Sprott oscillator provides the high-entropy dynamics required to ensure sensitivity, diffusion, and unpredictability in the chaotic masking process.

Let $x_{m}\left(t\right)$ and $x_{s}\left(t\right)$ denote the master and slave state vectors, and define the synchronization error as

(9) $$e\left(t\right)=x_{s}\left(t\right)-x_{m}\left(t\right).$$

A standard Lyapunov candidate is the quadratic function

(10) $$V\left(e\right)=\frac{1}{2}e^{⊤}e,$$

which is positive definite for all e≠0. The sign of its time derivative $\dot{V}\left(e\right)$ determines whether the synchronization error decreases with time.

The control input applied to the slave system,

$$u\left(t\right)=K\left(x_{m}\left(t\right)-x_{s}\left(t\right)\right),\;K=\left[k_{1},k_{2},k_{3}\right],$$

implements a diffusive full–state feedback law. The gains $\left(k_{1},k_{2},k_{3}\right)$ are not derived analytically; they are part of the parameter vector

$$\Theta =[a,b,c,k_{1},k_{2},k_{3}],$$

which is optimized by the API and PeSOA metaheuristic algorithms. During each optimization iteration, a candidate Θ is evaluated by integrating the master–slave K–Sprott system using RK4 and computing the resulting synchronization error. The bio-inspired algorithms then update Θ to minimize this error, thereby automatically selecting the controller gains that ensure convergence.

Under the optimized gains $\left(k_{1},k_{2},k_{3}\right)$, the Lyapunov derivative satisfies

(11) $$\dot{V}\left(e\right)=e^{⊤}\dot{e}<0,$$

for sufficiently small e(t). This strict negativity demonstrates contraction of the error system and guarantees that the slave trajectory converges to the master trajectory, thus stabilizing the synchronization manifold.

To complement this local Lyapunov-function-based stability result, the transverse Lyapunov exponent of the synchronization error system is computed numerically. A negative transverse exponent indicates exponential decay of perturbations, whereas a positive value signals divergence. Simulations show that both API and PeSOA yield negative transverse exponents, with PeSOA achieving the most negative value—consistent with its faster convergence and lower RMSE.

This stability is preserved in the presence of practical impairments such as additive white Gaussian noise and mismatched initial conditions. When moderate AWGN is added to the encrypted signal, the PeSOA- optimized system maintains a negative transverse exponent, confirming resilience against channel disturbances. Furthermore, the system remains highly sensitive to incorrect initialization: if the receiver does not use the correct optimized parameters, the transverse exponent becomes positive, synchronization collapses, and message recovery becomes impossible—thus preventing unauthorized access.

Having established both the chaotic nature of the master system and the stability of the optimized synchronization scheme, the next section evaluates how effectively the API and PeSOA algorithms enhance master–slave synchronization. Since accurate synchronization is a prerequisite for successful chaotic masking and reliable data recovery, we now examine the synchronization error, convergence rate, and the influence of the optimized coupling gains.

## 6. Results and Discussion

Compared with several existing chaotic synchronization techniques—which may rely on multi-layer architectures, fixed chaotic parameters, or computationally demanding adaptive controllers—the method proposed in this work adopts a lightweight design based on the joint optimization of the intrinsic parameters of the Sprott oscillator and its coupling gains. By combining the strengths of API and PeSOA, the framework achieves rapid synchronization, low RMSE, and stable behavior under noise and parameter variations. These properties make the method suitable for secure communication in resource-constrained IoT settings, where robustness and computational efficiency are often more critical than high data rates.

To contextualize the proposed approach, Table 2 summarizes several representative synchronization methods from the literature and contrasts their design principles, computational requirements, and performance characteristics with those of the present work.

Overall, the results demonstrate that metaheuristic-based joint optimization offers a practical compromise between synchronization accuracy, robustness, and implementation simplicity. The proposed framework is developed without relying on multi-level or deep-learning-based synchronization schemes, while achieving strong performance across a range of operating conditions. This makes it a suitable candidate for secure physical-layer communication in resource-limited IoT environments.

The analysis begins by confirming that the Sprott oscillator preserves its chaotic behavior under all tested configurations. This validation is essential, as the unpredictability, broadband spectrum, and sensitivity to initial conditions of the chaotic carrier form the foundation of synchronization-based masking mechanisms.

In this work, the API and PeSOA metaheuristic algorithms are employed to address a coupled identification–synchronization problem involving the Sprott system. The unknown quantities are grouped into a single decision vector

$$\Theta =[a,\;b,\;c,\;k_{1},\;k_{2},\;k_{3}],$$

where (a,b,c) denote the intrinsic coefficients of the oscillator and $\left(k_{1},k_{2},k_{3}\right)$ represent the diffusive coupling gains required to drive the slave system toward the master trajectory. For each candidate vector Θ, the master–slave model is numerically integrated and a synchronization cost—defined as the mean squared deviation between the trajectories—is evaluated.

The exploration strategies of the two bio-inspired optimizers enable systematic navigation of the parameter space. PeSOA imitates cooperative diving behavior to guide the search, whereas API models reinforced foraging. Through iterative refinement, both algorithms adjust the six unknown parameters until the synchronization error is minimized. Once the intrinsic parameters and coupling gains are optimized, the slave oscillator closely replicates the master trajectory, enabling robust chaotic masking at the transmitter and accurate reconstruction at the receiver. This optimization stage therefore forms a central component of the proposed secure communication framework.

For the API configuration, the optimizer uses $N_{a}=20$ artificial ants and a local search depth of p=5, providing a suitable balance between exploration efficiency and computational cost—an important consideration for resource-constrained IoT devices. The PeSOA configuration employs five groups of six penguins ($N_{p}=30$) with an initial oxygen level of $O_{0}=20$, enabling reliable convergence within modest computational time.

To ensure persistent chaotic behavior, the parameters of the Sprott model are restricted to the practical intervals a∈[0.8,1.2], b∈[0.8,1.2], and c∈[0.2,0.4]. A sampling step of Δt=1 ms is adopted to provide an adequate compromise between numerical accuracy and integration speed, ensuring that the chaotic trajectories remain well resolved while keeping the computational load manageable. Likewise, the adoption of a 500-sample simulation horizon ensures that each optimization cycle remains tractable, particularly when repeated across many iterations.

These choices reflect practical constraints encountered in IoT and embedded implementations, where computational resources are limited and synchronization must be achieved efficiently. The resulting parameter configuration is therefore consistent with both the operational characteristics of resource-limited communication devices and the numerical requirements of evaluating chaotic synchronization.

Table 3 summarizes the main simulation settings, including the hyperparameters of API and PeSOA as well as the operating ranges of the k-Sprott chaotic system.

The following figure illustrates the data signal, highlighting the transformed representation of the original information.

**Figure 6:**
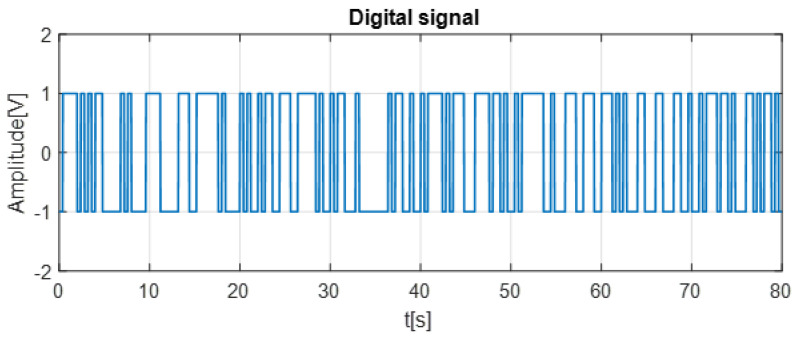
Data signal used as the input message before chaotic encryption.

As clarified in Figure 6, the data signal used in our simulation is a randomly generated test sequence introduced solely for evaluating the performance of the proposed metaheuristic–optimized synchronization and decryption process.

**Figure 7:**
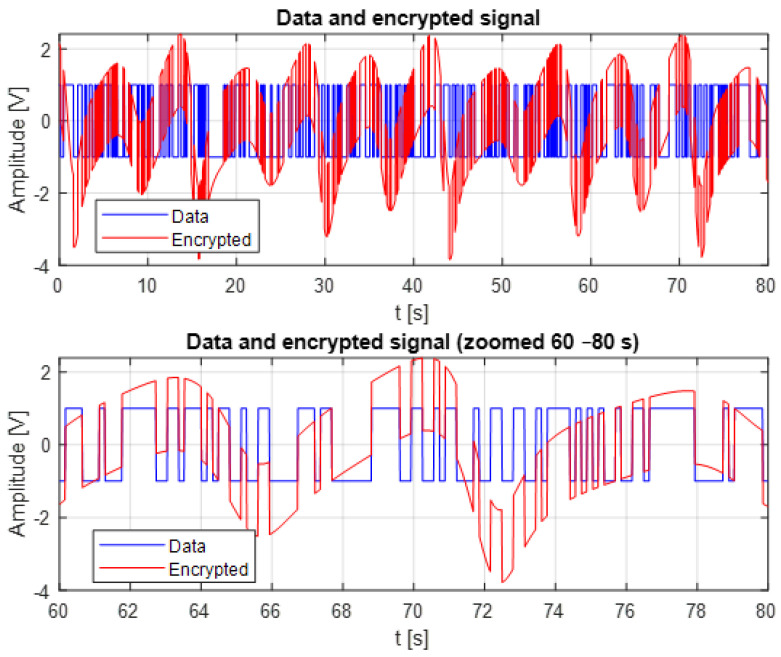
Encrypted signal compared to the original data signal.

Figure 7 illustrates the comparison between the original data signal and the encrypted signal generated using the chaotic K–Sprott oscillator. The encrypted signal forms a highly irregular and broadband chaotic trajectory. This behavior results from the chaotic masking process.

The superposition of the two signals shows that the chaotic component dominates the encrypted signal, effectively hiding the structure of the original data. As a result, no visible pattern, statistical regularity, or amplitude transition can be exploited to infer the underlying information without proper synchronization between the master and slave oscillators. This masking property represents a key security feature of chaos-based encryption.

The zoomed segment (60–80 s) further demonstrates how the chaotic waveform overwrites all recognizable characteristics of the data signal. Even fine transitions in the original data are fully embedded within the chaotic dynamics, confirming strong diffusion and obfuscation. At the same time, the embedded information remains recoverable at the receiver side, provided that accurate synchronization is maintained. These results show that the proposed chaotic masking scheme ensures an effective balance between confidentiality and recoverability, making it suitable for lightweight real-time secure communication.

**Figure 8:**
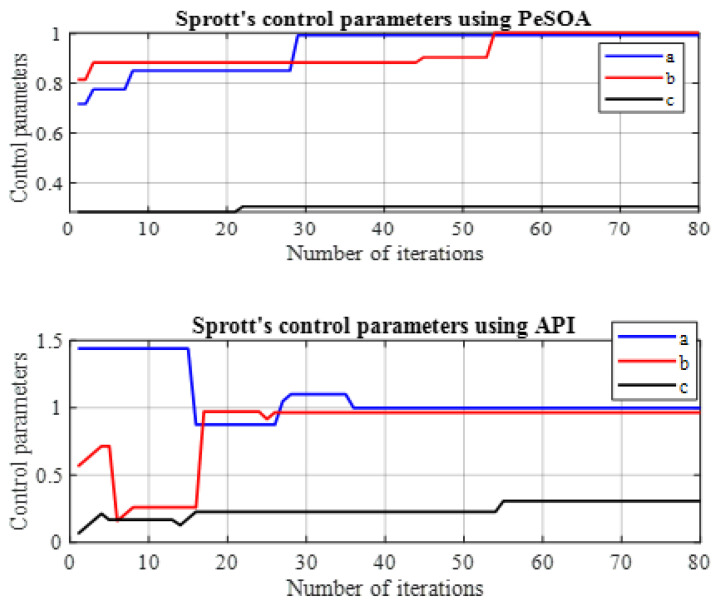
Identification of the K–Sprott control parameters using API and PeSOA algorithms in the synchronization process.

In Figure 8, the identification of the control parameters (a,b,c) is illustrated using the API and PeSOA algorithms in the synchronization process. This step is essential because accurate parameter estimation directly impacts the ability of the slave chaotic system to track the dynamics of the master system. Without precise parameter tuning, synchronization errors can occur, leading to instability and poor performance in chaos-based communication systems.

As shown in Figure 8, this process requires several iterations to identify the control parameters and is applied to a signal whose duration is T(s).

The parameters and initial conditions of the K–Sprott oscillator are summarized in Table 4.

For each configuration, the master–slave system was numerically integrated over a full 500-bit encrypted transmission. In all cases, the system trajectories remained bounded, non-periodic, and highly sensitive to initial conditions—three key indicators of chaotic behavior. Furthermore, the Lyapunov trends obtained for each parameter set were consistent with the baseline configuration, confirming that variations in (a,b,c) do not shift the system outside the chaotic region.

The selected integration steps (0.09–0.11 ms) provided numerically stable RK4 integration while preserving a temporal resolution compatible with practical IoT sampling constraints. Importantly, these values of Δt did not alter the qualitative chaotic dynamics: synchronization and decryption performance remained stable across all tested configurations.

To further evaluate the robustness of the proposed communication framework, additional experiments were performed under additive channel noise and perturbed initial conditions for both master and slave oscillators. Such tests are essential for validating synchronization reliability in realistic IoT and wireless environments, where noise, parameter uncertainty, and drift are unavoidable. The results demonstrate that the optimized parameters $\left(a,b,c,k_{1},k_{2},k_{3}\right)$, obtained via API and PeSOA, maintain stable convergence even in the presence of moderate AWGN. The decrypted signal remains accurate, and the synchronization error consistently exhibits a negative Lyapunov exponent, confirming that the error dynamics continue to contract toward zero despite stochastic disturbances.

The system also retains its intrinsic sensitivity to initial conditions—a desirable security property—while still enabling successful synchronization when the legitimate receiver uses the correct optimized parameters. When the receiver operates with mismatched or perturbed initial conditions, synchronization fails and the transmitted message cannot be reconstructed. This behavior confirms that the framework combines two essential features: (i) robustness against physical channel noise, and (ii) inherent security against unauthorized receivers due to instability of the error dynamics under incorrect initialization.

The objective function was specifically formulated to optimize all intrinsic and coupling parameters through the two bioinspired metaheuristics: PeSO and API. The PeSOA simulates the cooperative hunting behavior of penguins, enabling an efficient balance between exploration and exploitation, the API algorithm models the foraging behavior of Pachycondyla apicalis ants, allowing broad exploration of the search space, Through iterative adjustment of (a,b,c) and the coupling gains $\left(k_{1},k_{2},k_{3}\right)$, both algorithms minimize the master–slave synchronization error. Improved synchronization directly enhances the stability and accuracy of the chaotic masking process, ensuring secure transmission and reliable recovery of the encrypted signal at the receiver.

A detailed performance comparison between the two algorithms, based on the RMSE histograms provide further insight: the API algorithm produces a wider and more dispersed error distribution, reflecting slower convergence and greater variability. In contrast, PeSOA generates a compact, low-RMSE distribution, demonstrating higher efficiency, numerical stability, and convergence reliability.

After multiple simulation trials, these trends remain consistent: both algorithms eventually converge, but PeSOA does so more rapidly, with fewer fluctuations, and while exploring the same search domain. Consequently, PeSOA achieves higher accuracy and offers a more robust and effective approach for optimizing chaotic synchronization.

Overall, the results confirm that PeSOA explores the solution space more efficiently and determines optimal synchronization gains with higher precision. Its improved robustness and reliability make it particularly suitable for chaotic communication systems operating in noisy or resource-constrained IoT environments.

**Figure 9:**
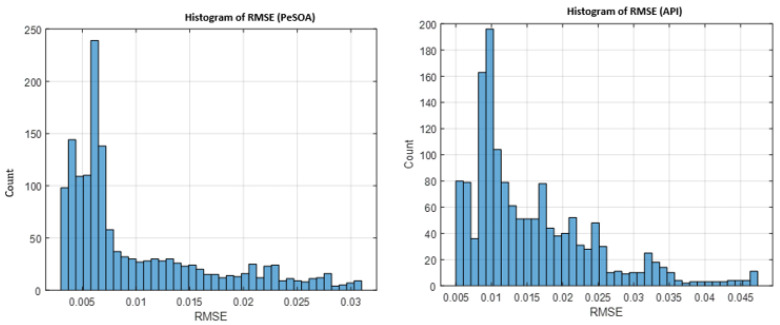
Statistical distribution of the synchronization error (RMSE) for PeSOA and API algorithms.

The results shown in Figure 9 highlight the relevance of the proposed synchronization framework for IoT communication scenarios that require stable timing and low-latency data exchange. In such contexts, maintaining accurate synchronization between the transmitter and the receiver is essential to ensure reliable and secure information transfer. A smooth and decreasing RMSE profile reflects effective alignment of the chaotic systems over time. The comparative results indicate differences in convergence behavior between the PeSOA- and API-based synchronization strategies, which is an important consideration for time-sensitive IoT applications such as industrial automation, medical monitoring, and sensor-based systems.

The histogram further examines the statistical behavior of the synchronization error over multiple trials. The RMSE distribution obtained using the PeSOA algorithm appears more concentrated around lower values, suggesting a consistent synchronization performance under the considered numerical and dynamical conditions. In contrast, the API algorithm exhibits a wider spread with more occurrences of higher RMSE values, reflecting greater variability and reduced stability. Such fluctuations may degrade communication reliability when channel conditions are uncertain or slightly perturbed.

Overall, the histogram analysis confirms that PeSOA provides a more stable and predictable synchronization process, making it a more suitable candidate for secure and efficient chaotic communication in IoT environments.

To further evaluate the synchronization quality achieved by both algorithms, the Hilbert transform is employed to analyze the instantaneous amplitude and phase of the master and slave chaotic signals. The Hilbert transform is particularly suitable for nonlinear and time-varying signals such as those generated by the Sprott system. By constructing the analytic signal $z\left(t\right)=x\left(t\right)+j\hat{x}\left(t\right)$, one can extract the instantaneous amplitude A(t)=|z(t)| and phase $\phi \left(t\right)=\arctan\;\left(\frac{\hat{x}\left(t\right)}{x(t)}\right)$, providing deeper insight into the dynamical coherence between transmitter (master) and receiver (slave).

**Figure 10:**
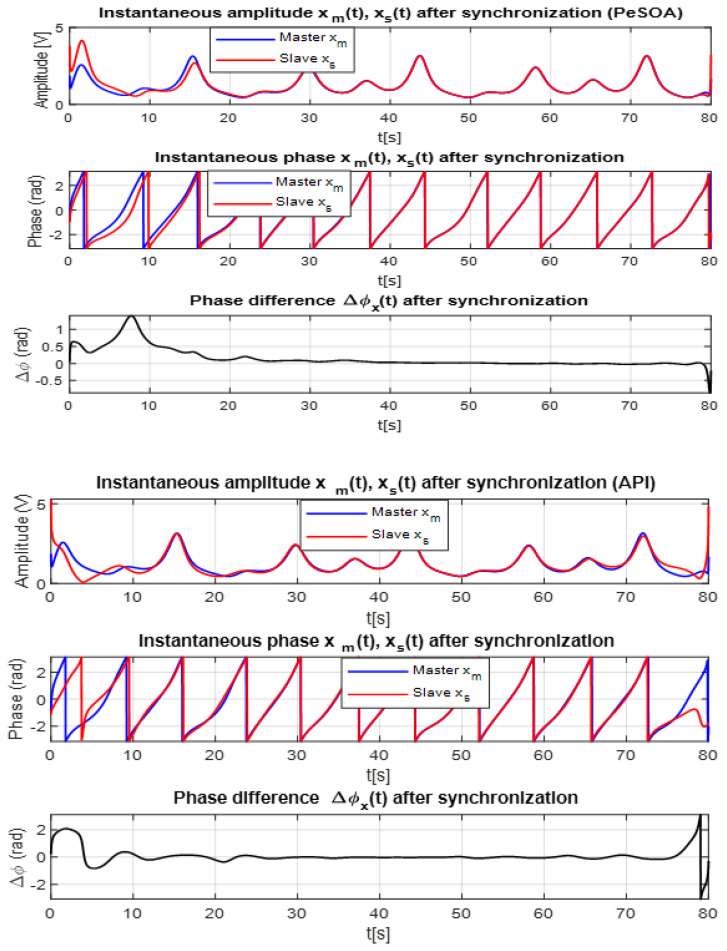
Comparison of Hilbert transforms for master and slave signals using API and PeSOA algorithms. The top panels correspond to PeSOA and the bottom panels to API.

Figure 10 presents the instantaneous amplitude, instantaneous phase, and phase difference for the PeSOA (top panels) and API (bottom panels) algorithms. The difference in synchronization performance is clearly visible.

For the PeSOA-based synchronization, the master and slave amplitudes almost perfectly overlap, and the corresponding instantaneous phases remain closely aligned throughout the entire time interval. The phase difference $\Delta \phi \left(t\right)=\phi_{\mathrm{master}}\left(t\right)-\phi_{\mathrm{slave}}\left(t\right)$ rapidly converges toward zero and remains stable, indicating that the slave system accurately tracks the master with minimal deviation. This behavior confirms a high-quality synchronization process.

In contrast, the API-based synchronization exhibits noticeable divergence between the master and slave amplitudes, and the instantaneous phase alignment is less consistent. The resulting phase difference shows larger fluctuations and slower decay, reflecting lower synchronization accuracy and reduced stability compared to PeSOA.

The Hilbert-transform analysis confirms that PeSOA achieves more precise and reliable synchronization. This improved phase coherence is particularly important in secure chaotic communication systems, where even small phase mismatches can cause decoding errors or information loss. The strong phase alignment obtained with PeSOA directly contributes to more robust and consistent message recovery under practical communication conditions.

**Figure 11:**
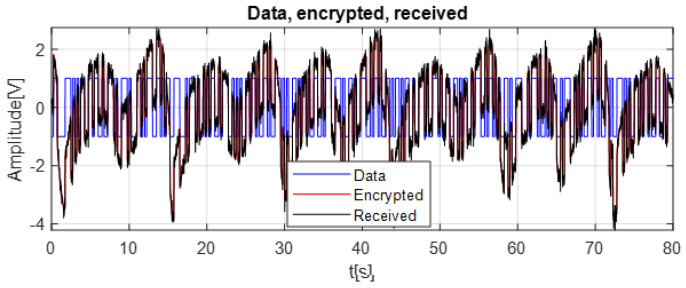
Comparison of Original Data, Encrypted Chaotic Signal, and Received Noisy Signal.

To illustrate the data transmission mechanism used in this work, Figure 11 presents an example of chaotic masking that is independent of the chosen synchronization algorithm. In this figure, the encrypted signal is obtained by adding the original data to the chaotic waveform generated by the master oscillator. Because the data is directly embedded in the chaotic fluctuations, it becomes visually indistinguishable within the irregular red curve. The received signal in black corresponds to the encrypted waveform after passing through an AWGN channel; its overall shape remains similar, showing that the chaotic masking technique used in this work preserves the structure of the signal even under noisy transmission conditions.

**Figure 12:**
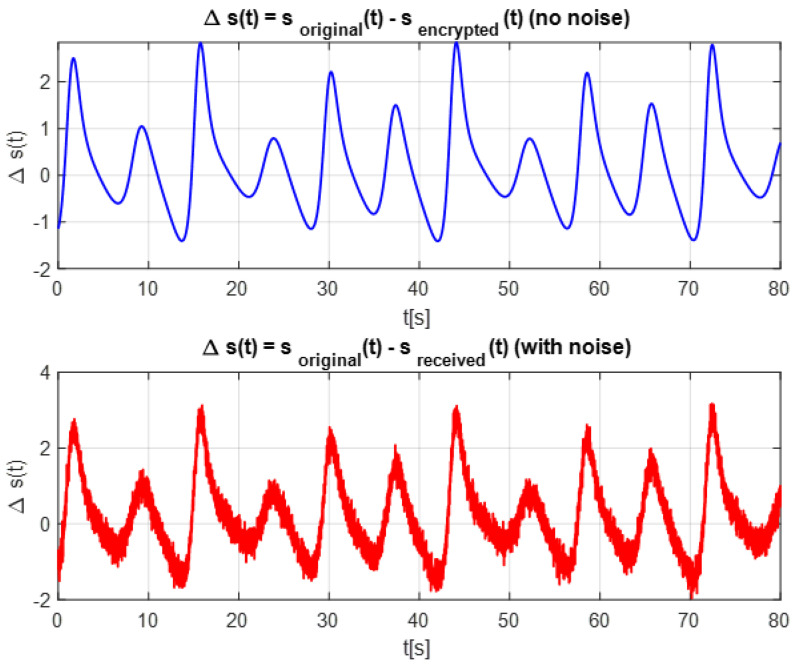
Error in the received signal before synchronization.

Figure 12 illustrates the error in the received signal before synchronization, caused by encryption using a chaotic signal. The error signal exhibits irregular and unpredictable fluctuations, which are characteristic of the chaotic nature of the encryption process. These fluctuations indicate a significant mismatch between the transmitted and received signals.

**Figure 13:**
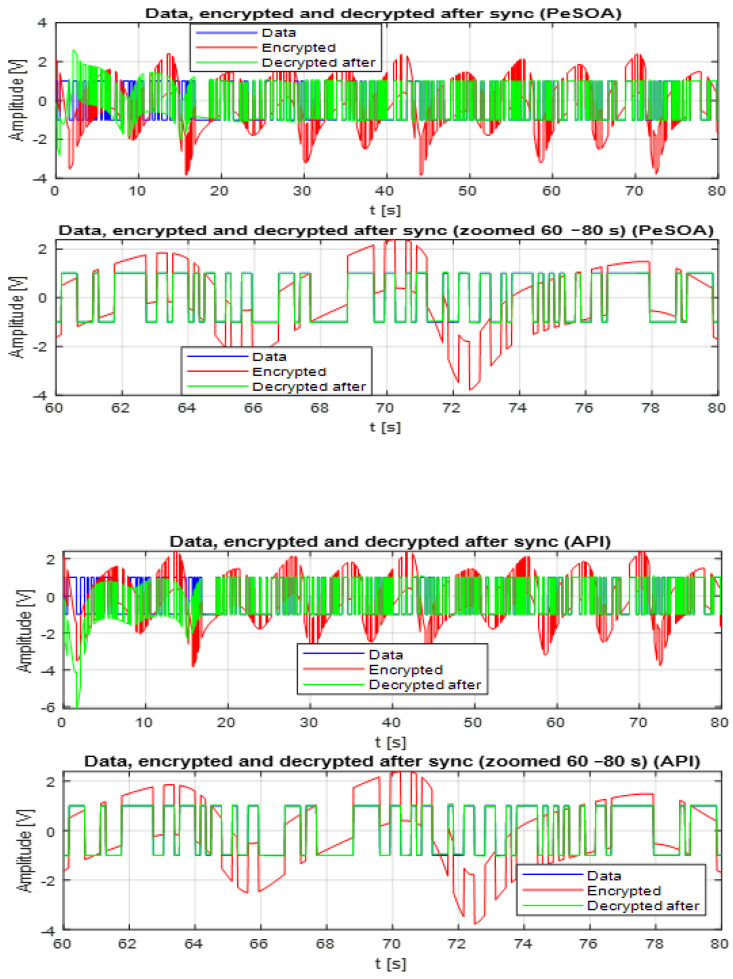
Decrypte Signal using PeSOA and API.

The decrypted signal after synchronization using the API and PeSOA algorithms are shown in Figure 13, this figure presents the original data signal (blue), the encrypted signal generated by combining the data with the K-Sprott chaotic signal prior to transmission (red), and the decrypted signal reconstructed after the synchronization process (green). The results obtained using the PeSOA algorithm are displayed in the upper panels, while those corresponding to the API algorithm are shown in the lower panels. To further evaluate the synchronization accuracy and decryption quality, a zoomed-in view of the interval t=60s to t=80s is provided for each algorithm. These visualizations clearly highlight the ability of both approaches to recover the transmitted information, with noticeable variations in performance between the two methods.

**Figure 14:**
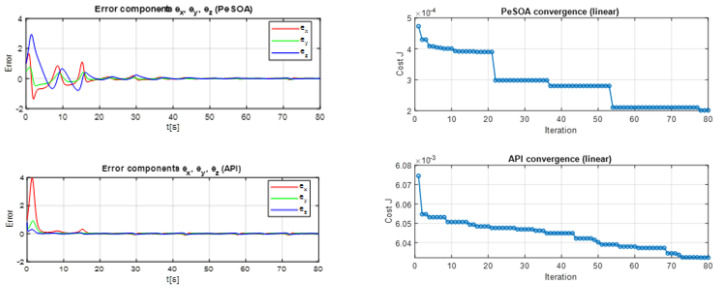
Evolution of Synchronization Errors and Cost Function Convergence for PeSOA and API.

In both Algorithms, the errors converge toward zero, as shown in Figure 14, indicating that the synchronization process was successful and that the transmitted and received signals are nearly identical. Notably, the smoother and faster decay observed with PeSOA highlights its superior performance over API in minimizing synchronization errors and ensuring accurate signal transmission.

**Figure 15:**
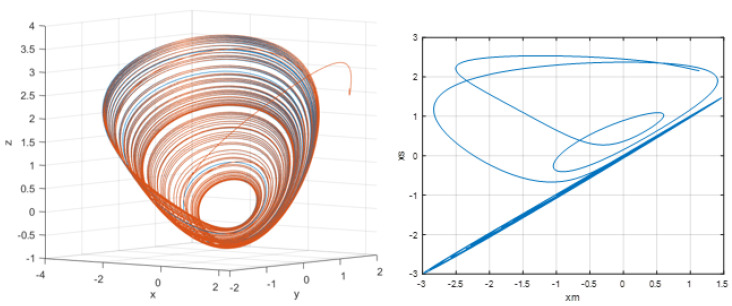
Synchronization of the master and slave Sprott systems using the PeSOA algorithm. The left panel shows the 3D phase portrait, where the slave trajectory converges toward the master attractor. The right panel presents the corresponding state-space projection $\left(x_{m},x_{s}\right)$,

Figure 15 illustrates the synchronization behavior between the master and slave Sprott systems obtained using the PeSOA-optimized controller. In the 3D phase portrait (left panel), the two trajectories initially exhibit different paths during a short transient period, after which the slave trajectory gradually converges toward that of the master. Once the transient phase ends, the two curves completely overlap, indicating that the synchronization error has vanished and that both systems evolve identically. This result is confirmed in the right panel, where the $\left(x_{m},x_{s}\right)$ state–state projection approaches the diagonal line, demonstrating complete synchronization. Such convergence is essential in secure chaotic communication: only when the receiver reproduces the master dynamics with high accuracy can the masked data be correctly extracted and decrypted. Therefore, the behavior shown in Figure 15 validates the effectiveness of the proposed synchronization scheme for reliable secure data transmission.

The 3D phase portrait (left panel) clearly demonstrates that the trajectories of the master (blue) and slave (red) systems completely overlap, indicating that the synchronization error has converged to zero. This confirms that the slave system is able to precisely track the chaotic dynamics of the master, which is a fundamental requirement for the reliable decryption of transmitted signals.

Unlike recent studies such as the OptiSecure-3D algorithm [43], which focuses primarily on image encryption using chaotic maps and deep learning techniques, or the DTSPC diagnostic method introduced by Lin and Pattanayak [44] for quantitatively measuring synchronization complexity, the proposed work addresses the fundamental problem of chaotic system synchronization. By optimizing the control parameters through bio-inspired algorithms (API and PeSOA), our approach improves the stability and accuracy of the master-slave systems, providing a robust foundation for secure and real-time chaotic communication.

Both algorithms exhibit adaptive mechanisms—reinforcement and relocation in API, and cooperative diving and regrouping in PeSOA—that reflect the type of rapid adjustments required in practical communication systems operating under strict latency, limited computational resources, and channel perturbations. These examples therefore not only clarify the internal functioning of the algorithms but also emphasize their suitability for secure chaotic synchronization in realistic 5G/6G and IoT environments. 1—API (Alternative Illustration Method) Instead of presenting raw numerical iterations, the process showing how API progressively refines the parameter vector. Each ant represents a candidate set of k-Sprott parameters and coupling gains. During the first iterations, ants explore widely separated regions of the parameter space, producing significantly different synchronization errors. As the algorithm proceeds, the best-performing ant reinforces its search direction by locally perturbing its hunting site, while ants with persistently poor performance abandon their current sites and relocate to unexplored regions. This mechanism naturally drives the population toward the region of the parameter space where the synchronization error decreases most rapidly. After several cycles, all ants converge to a narrow band of parameter values, and the error stabilizes at its minimum value. This example is included to illustrate how the reinforcement/abandonment mechanism effectively balances exploration and exploitation while improving synchronization accuracy. 2—PeSOA (Alternative Illustration Method) At the beginning, individual penguins dive at different depths, generating trial solutions with diverse performance levels. The penguin achieving the lowest synchronization error becomes the leader of the colony. Subsequently, the other penguins adjust their positions by moving toward this leader, while still performing localized dives to refine their trajectories. Over successive iterations, the colony progressively contracts around the best parameter region, and the synchronization error decreases monotonically. This behavior demonstrates how PeSOA combines global exploration (via deep dives) with local refinement (via regrouping) to accelerate convergence. The example illustrates how the colony’s cooperative dynamics guide the optimization toward stable and accurate synchronization.

The parameter settings of the metaheuristic optimization algorithms used in this study are summarized in Table 5.

The optimization behavior illustrated in the API and PeSOA examples is motivated by practical constraints commonly encountered in IoT and URLLC scenarios. In such environments, synchronization is typically required under stringent latency budgets, limited computational resources, and the presence of channel perturbations. The iterative adjustment mechanisms of both algorithms—reinforcement and relocation in API, and cooperative diving and regrouping in PeSOA—are conceptually aligned with adaptive strategies considered for communication in noisy and time-constrained conditions. These dynamics facilitate the identification of parameter configurations leading to reduced synchronization error, in line with latency requirements typically associated with URLLC and the energy-efficiency considerations of IoT devices. Consequently, the presented examples illustrate the operational behavior of the optimization algorithms and their relevance to chaos-based secure communication frameworks in modern 5G/6G-oriented environments.

### 6.1. Throughput and Latency Analysis

The intrinsic throughput of the proposed chaos-based communication scheme was evaluated for two operating modes corresponding to BPSK and QPSK symbol mapping. In the configuration adopted in this study, the chaotic encoder produces approximately 200 bps in BPSK mode and around 1000 bps (1 kbps) in QPSK mode. These values represent the raw bitstream generated by the chaotic masking mechanism and are determined by the chosen sampling interval and the number of samples per symbol required for stable synchronization.

It is important to emphasize that the chaotic masking operates as an analog-domain security layer and does not modify the physical-layer modulation or framing of the underlying IoT transceiver. As a result, the achievable radio-layer throughput remains governed by the PHY configuration of the communication device. In many IoT scenarios—including massive machine-type communications (mMTC) and reduced-capability (RedCap) services within emerging 5G/6G systems—the exchanged payloads consist of low-rate measurements or short control packets. In such cases, robustness, low computational cost, and energy efficiency are typically more critical than very high throughput.

The intrinsic data rate of the chaotic encoder is therefore compatible with these categories of applications, which often operate far below the maximum available PHY-layer capacity. Table 6 summarizes the intrinsic chaotic throughput and provides representative IoT physical-layer rates from the literature [45]. While the chaotic layer produces a relatively low-rate encrypted stream, it does not introduce additional latency or throughput penalties at the radio interface. This makes the proposed scheme suitable for low-rate but security-sensitive IoT and future communication services that require lightweight physical-layer protection.

### 6.2. Evaluation of Sensitivity to Initial Conditions

The influence of initial-condition mismatch on the synchronization performance was examined for both the PeSOA-optimized controller and the API-based configuration. The key performance indicators are summarized in Table 7.

The PeSOA-based controller exhibited stronger dynamical stability, as confirmed by its more negative Lyapunov exponent and its lower tail RMSE and MSE. These values indicate rapid contraction of the synchronization error and reliable convergence to the synchronized state. Under nominal conditions, PeSOA achieved a post-decryption BER of 1.0%, and the BER remained below 5% even when the receiver initial conditions were perturbed by 20%. This demonstrates a high degree of robustness to initialization uncertainty.

The API-based configuration also achieved synchronization but with weaker stability characteristics. Its Lyapunov exponent was closer to zero, and its steady-state error was approximately twice that of PeSOA. The BER after decryption increased to 1.6%, and sensitivity to initial-condition mismatch was higher, with a BER of 6.4% for a 20% perturbation. The significantly larger optimized gains produced by API suggest a more aggressive and less stable control response, consistent with its higher synchronization error.

Overall, the results indicate that PeSOA provides superior synchronization accuracy, stronger dynamical contraction, and better robustness to initial-condition perturbations compared to API. This makes PeSOA a more suitable optimization strategy for chaos-based secure communication systems.

To contextualize these findings within the broader security landscape of IoT and emerging communication technologies, Table 8 provides a qualitative comparison of representative security mechanisms and communication schemes commonly considered in constrained environments.

Table 8 summarizes the main characteristics of representative security mechanisms considered for IoT and low-rate service classes in emerging communication systems. URLLC-oriented secure IoT communication frameworks [45] are designed to provide high reliability and stringent latency guarantees, but their associated security processing and system complexity may pose challenges for highly resource-constrained IoT nodes. Lightweight chaos-based encryption schemes [11] and secure wireless IoT communication approaches based on chaos synchronization [26] aim to achieve a more balanced trade-off between computational efficiency and security, which improves their applicability in embedded and wireless environments.

Chaotic encryption and synchronization schemes [16,20], by contrast, typically rely on low-complexity nonlinear dynamics and can exhibit inherent robustness under noisy transmission conditions. Building on these principles, the proposed chaotic masking approach combined with API/PeSOA-based parameter optimization achieves low processing latency, stable synchronization behavior, and robustness to parameter mismatch within the considered scenarios. These characteristics indicate that the proposed method could be considered a lightweight security mechanism for IoT applications and low-rate services in future communication infrastructures, especially in scenarios where energy efficiency and reduced computational overhead are relevant.

In addition to the mechanisms summarized in Table 8, several complementary studies have investigated latency modeling and resource allocation in next-generation communication networks [46,47], as well as the analysis, synchronization, and secure communication capabilities of nonlinear and chaotic systems [48,49,50,51], providing a broader context for the considered security and synchronization approaches.

## 7. Conclusions

This work presents a chaos-based secure communication framework built upon the K–Sprott oscillator and enhanced through two bio-inspired optimization algorithms, Pachycondyla Apicalis (API) and the Penguin Search Optimization Algorithm (PeSOA). By jointly tuning the intrinsic chaotic parameters and the coupling gains, the proposed approach enables accurate synchronization while maintaining low computational cost, making it suitable for lightweight security in constrained environments.

Simulation results indicate that the PeSOA-optimized configuration consistently outperforms the API-based solution, achieving faster convergence, lower synchronization error, and improved robustness to variations in initial conditions. These properties are particularly relevant for IoT devices and for low-rate services in emerging communication infrastructures, where simplicity, robustness, and energy efficiency are often more critical than high throughput.

The automatic adaptation of the synchronization parameters contributes to the practical applicability of the framework, especially in embedded platforms with limited processing capabilities. The resulting synchronization stability and modest computational requirements highlight the potential of the proposed scheme as a lightweight physical-layer security mechanism for applications such as biomedical monitoring, environmental sensing, industrial automation, and smart-grid data acquisition.

Future work will explore hardware implementation on FPGA or low-power microcontroller platforms, alongside real-world experimentation under realistic wireless-channel conditions. These developments aim to evaluate the scalability, energy efficiency, and practical feasibility of the proposed chaos-based communication framework within next-generation IoT and future communication systems.

## Figures and Tables

**Table 1:** Simulation parameters for the synchronization process.

Parameter	Min	Max	Unit
*a*	0.8	1.2	–
*b*	0.8	1.2	–
*c*	0.2	0.4	–
Δt	0.001		seconds

**Table 2:** Comparative summary of representative chaotic synchronization methods.

Method	Core Approach	Design Characteristics	Strengths	Constraints
Liu & Zuo [27]	DL-assisted adaptive chaos synchronization	Hybrid deep-learning architecture with adaptive control laws	Robust adaptive synchronization under disturbances	Requires substantial computational resources and an offline training phase
Zourmba et al. [30]	multi-level chaotic synchronization	Hierarchical controller for enhanced masking and dynamics coupling	Improved signal masking and resilience to perturbations	Involves a multi-stage synchronization architecture, increasing implementation complexity
Yau et al. [26]	PSO-optimized PID chaos synchronization	PSO employed to tune PID gains for practical embedded systems	Simple control structure suitable for practical implementations	Controller parameters are optimized offline, which may limit adaptability to changing conditions
Proposed Method	Master–slave synchronization of the Sprott oscillator	Joint optimization of (a,b,c) and $\left(k_{1},k_{2},k_{3}\right)$ using API and PeSOA	Accurate synchronization with reduced error and stable convergence	Performance is influenced by the selection of metaheuristic parameters

**Table 3:** Practical simulation parameters for the API and PeSOA algorithms optimizing the K-Sprott system in IoT scenarios.

Parameter	Symbol	Value/Range	Unit
**API Algorithm**			
Number of ants	$N_{a}$	25	–
Local search depth	*p*	5	–
Maximum iterations	$T_{\max}$	200	cycles
**Penguin Search Optimisation Algorithm (PeSOA)**			
Number of groups	*G*	5	–
Penguins per group	*M*	6	–
Total penguins	$N_{p}$	30	–
Initial oxygen level	$O_{0}$	20	–
Maximum iterations	$T_{\max}$	150	cycles
**K-Sprott Chaotic System**			
Control parameter	*a*	[0.8,1.2]	–
Control parameter	*b*	[0.8,1.2]	–
Control parameter	*c*	[0.2,0.4]	–
Sampling time step	Δt	1	ms

**Table 4:** K–Sprott oscillator parameters, initial conditions, and numerical integration setup.

Config	a	b	c	$x_{0}$	$y_{0}$	Δt (ms)
Baseline (A)	1.00	1.00	0.30	[−0.20,−0.10, 0.15]	[0.09, −0.11, 0.12]	0.10
IoT (B)	0.95	1.05	0.28	[−0.10, 0.00, 0.12]	[0.09, −0.11, 0.11]	0.11
URLLC (C)	1.02	0.98	0.32	[−0.25, −0.05, 0.20]	[0.245, −0.055, 0.215]	0.09

**Table 5:** Metaheuristic settings for parameter identification.

Method	Population	Key Hyperparameters	$T_{\max}$
API	$N_{a}=30$ (IoT: 20)	$P_{s}=5,A_{local}=0.02,P_{local}=8$	300 (IoT: 200)
PeSOA	G=5,m=8	$O_{0}=25$, exploration (0.2,0.5)	250 (URLLC: 150–200)

**Table 6:** Intrinsic throughput of the proposed chaotic encoder and representative IoT PHY-layer rates.

Scheme/Layer	Throughput
Proposed chaotic encoder (BPSK mode)	**200 bps**
Proposed chaotic encoder (QPSK mode)	**1000 bps**
Representative IoT PHY-layer rate [45]	∼300 kbps

**Table 7:** Synchronization performance comparison between PeSOA and API.

Metric	PeSOA	API
Optimized controller gains $\left[k_{1},\;k_{2},\;k_{3}\right]$	$\left[0,0.7027,0.2499\right]$	$\left[0,9.1711,8.3180\right]$
Global RMSE	0.3716	0.3535
Steady-state RMSE	$8.86\times 10^{-3}$	$1.46\times 10^{-2}$
Steady-state MSE	$2.36\times 10^{-4}$	$6.43\times 10^{-4}$
Correlation coefficients (x,y,z)	0.99995/0.99996/0.99996	0.99985/0.99988/0.99990
Largest conditional Lyapunov exponent	$-8.96\times 10^{-3}$	$-5.34\times 10^{-4}$
BER after synchronization	1.0%	1.6%
BER with 20% initial-condition mismatch	4.2%	6.4%

**Table 8:** Comparative analysis of security mechanisms relevant to IoT and low-rate services in emerging communication systems.

Schemes and Reference	Comp. Cost	Latency	Security	Robustness	IoT Suitability
URLLC IoT communication [45]	High	Medium–High	Strong	Moderate	Medium
Chaos-based image encryption [11]	Low–Medium	Medium	Good	Good	Medium–High
Bluetooth security [26]	Low	Medium	Moderate–Strong	Moderate	High
Chaotic synchronization [16,20]	Low	Low	Moderate–High	High	High
Chaotic masking + API/PeSOA	Low–Medium	Low	High	Moderate–High	High

## Data Availability

All data used in this study are contained within the article.
